# NAD+ biosynthesis metabolism predicts prognosis and indicates immune microenvironment for breast cancer

**DOI:** 10.3389/pore.2023.1610956

**Published:** 2023-03-17

**Authors:** Yuting Yang, Ze Wang, Mengqi He, Lihong Diao, Biyue Yu, Dong Li

**Affiliations:** ^1^ Department of Immunology, Medical College of Qingdao University, Qingdao, Shandong, China; ^2^ State Key Laboratory of Proteomics, Beijing Proteome Research Center, National Center for Protein Sciences, Beijing Institute of Lifeomics, Beijing, China; ^3^ School of Traditional Chinese Medicine, Beijing University of Chinese Medicine, Beijing, China; ^4^ School of Life Sciences, Hebei University, Baoding, Hebei, China

**Keywords:** immunotherapy, breast cancer, prognosis, immune microenvironment, NAD+ biosynthesis

## Abstract

The growing evidence implies that tumor cells need to increase NAD+ levels by upregulating NAD+ biosynthesis to satisfy their growth demand. NAD+ biosynthesis metabolism is implicated in tumor progression. Breast cancer (BC) is the most common malignant malignancy in the world. Nevertheless, the prognostic significance of NAD+ biosynthesis and its relationship with the tumor immune microenvironment in breast cancer still need further investigation. In this study, we obtained the mRNA expression data and clinical information of BC samples from public databases and calculated the level of NAD+ biosynthesis activity by single-sample gene set enrichment analysis (ssGSEA). We then explored the relationship between the NAD+ biosynthesis score, infiltrating immune cells, prognosis significance, immunogenicity and immune checkpoint molecules. The results demonstrated that patients with high NAD+ biosynthetic score displayed poor prognosis, high immune infiltration, high immunogenicity, elevated PD-L1 expression, and might more benefit from immunotherapy. Taken together, our studies not only deepened the understanding of NAD+ biosynthesis metabolism of breast cancer but also provided new insights into personalized treatment strategies and immunological therapies to improve the outcomes of breast cancer patients.

## Introduction

Breast cancer (BC) is the most common malignancy in the world and the fifth leading cause of cancer death worldwide (1). With advance in the diagnosis and treatment of breast cancer, the survival rate of breast cancer patients has significantly improved (2). However, as a malignancy with high heterogeneity at the molecular level, BC patients with similar clinical features may have different prognosis (3). Therefore, it is essential to consider other important factors in guiding clinical practice.

The tumor microenvironment (TME) is a highly complex cellular network, including tumor cells, stromal cells, fibroblasts, immune cells, soluble factors, signaling molecules and extracellular matrix components (4). Recent studies suggest that the immune components in the TME are closely associated with tumor development, recurrence, and metastasis. These components are called the tumor immune microenvironment (TIME) (5). Immune cells or immune-related genes in TIME can predict the prognosis and treatment efficacy of cancer patients (6–8). For instance, tumor-infiltrating lymphocytes (TILs) have been shown to have a strong prognostic effect in patients with early stage TNBC and HER2-positive breast cancer (9, 10). Exploring TIME in BC would conductive to guide and optimize immunotherapy and improve the prognosis of BC patients.

NAD+ (nicotinamide adenine dinucleotide) is a coenzyme for redox reactions and a substrate for different signaling enzymes that can directly or indirectly affect many important cellular functions, such as metabolic pathways, DNA repair, and immune cell function (11, 12). Many of these processes are associated with cancer development. Given that NAD+ -dependent signaling responses involve the degradation of molecules, sustained NAD+ production *via* different biosynthetic pathways is a hallmark of many types of tumor (13, 14). Enzymes involved in NAD+ biosynthesis have been reported to be aberrantly expressed or dysregulated in many cancer types, including breast cancer. NAMPT, one of the rate-limiting enzymes of NAD+ biosynthesis, has been shown to be overexpressed in breast cancer and associated with breast cancer proliferation and invasiveness (15, 16). However, the relationship between NAD+ biosynthesis and the immune microenvironment of breast cancer has not been systematically investigated.

In the current study, a series of bioinformatic methods were used to analyze the features of NAD+ biosynthesis in BC based on transcriptional profiling data from public databases. BC patients were classified into the high and low NAD+ biosynthetic subtypes based on the NAD+ biosynthesis score and survival analysis was performed. We further explored the hallmark pathways and TME immune cell infiltration characteristics of the two subtypes. Moreover, we analyzed the relationship between the NAD+ biosynthesis score and several immunotherapy biomarkers. These findings may provide a new perspective for exploring the metabolic mechanism and treatment of breast cancer.

## Materials and methods

### Retrieval of NAD+ biosynthesis-related genes

NAD+ biosynthesis-related genes were obtained from the Kyoto Encyclopedia of Genes and Genomes (KEGG) pathway database (Pathway: hsa00760) and Reactome database (R-HSA-196807) (17). We also reviewed the literature and added the previously reported genes (11). After selecting genes associated with NAD+ biosynthesis from all gene sets, a total of 19 genes were retrieved (Supplementary Table S1).

### Datasets and data preprocessing

The mRNA sequencing, gene somatic mutations, and clinical data of breast invasive carcinoma (BRCA) patients were downloaded from The Cancer Genome Atlas (TCGA) database and included data form 99 normal samples and 1069 tumor samples. RNA-sequencing data (FPKM values) were transformed into transcripts per million (TPM) values and normalized into log2 (TPM +1) for the following analysis. Datasets of GSE20711, GSE48390 and GSE88770 from the Gene Expression Omnibus (GEO) were selected to validate the results of TCGA data analysis. The GSE20711 dataset was used by Dedeurwaerder et al. to study epigenetic variation (methylation) related to gene expression in breast cancer (18). GSE20711 consists of 88 breast cancer and 2 normal breast tissue samples, with only the gene expression profiles of 88 breast cancer patients retained for subsequent analysis. The GSE48390 dataset was used by Huang et al. to identify genes with coherent patterns of both copy number variation (CNV) and differential gene expression, and to use these genes to derive signatures related to clinical ER and HER2 status and disease-free survival (19). GSE48390 consists of 81 breast cancer samples. The GSE88770 dataset was used by Metzger-Filho et al. to analyze the prognostic value of histological grading (HG) in breast cancer (20). GSE88770 contains 117 breast cancer samples. The three datasets were based on GPL570 platforms (Affymetrix Human Genome U133 Plus 2.0 Array). We downloaded the original expression profile and used the robust multi-array average (RMA) algorithm to perform background correction and quantile normalization (21). We merged GEO datasets and used the combat algorithm to eliminate the batch effects by R package “sva” (22).

### Evaluation of tumor NAD+ biosynthesis score

We applied the single-sample gene-set enrichment analysis (ssGSEA) for the NAD+ biosynthesis gene set to quantify the NAD+ biosynthesis activity (NAD+ biosynthesis score) (23). The optimal cutoff threshold for classifying breast cancer (BC) patients into high and low NAD+ biosynthetic subtypes was determined using the “surv_cutpoint” function from the R package “survminer”.

### Differential gene expression analysis and enrichment analysis

We identified differentially expressed genes (DEGs) between the high and low NAD+ biosynthetic subtypes using the R package “limma,” with a significance threshold of adjusted *p*-value <0.05 and |log2FoldChange| > 1. To assess the differences in biological signaling pathway between the high and low NAD+ biosynthetic subtypes, GSEA analysis was performed using the gseKEGG function of the R package “clusterProfiler,” with *p* < 0.05 considered statistically significant (24).

### Immune infiltration analysis

Immune cell gene signatures and immune-related signatures were collected from previously published works (25, 26), and ssGSEA was used to calculate the enrichment scores of infiltrating immune cells and immune functions. In addition, the “ESTIMATE” package was used to assess the composition of the immune stroma in the tumor microenvironment of breast cancer patients, and the immune score, stromal score, and estimated score (ESTIMATE Score) were calculated (27).

### Construction of TF-IRG networks

We extracted differentially expressed immune-related genes (DEIRGs) and transcription factors (DETFs) from DEGs based on the lists obtained from ImmPort and Cistrome Cancer database (Supplementary Tables S2, S3). We then calculated the correlation between DETFs and DEIRGs. Correlation coefficients >0.5 (or less than −0.5) and adjusted *p*-value <0.01 were considered to be significantly correlated (Supplementary Table S4).

### Mutation and evaluation of the therapeutic efficacy

The R package “maftools” was used to estimate the tumour mutation burden (TMB) for each patient between the two subtypes (28). Additionally, the neoantigen load and homologous recombination deficiency (HRD) in BRCA patients were collected from published studies (29, 30). For the immunotherapy sensitivity prediction analysis, the immunophenoscore (IPS) of breast cancer patients was downloaded from the TCIA database (25). For drug sensitivity prediction analysis, the “pRRophetic” R package was used to calculate the IC50 of common chemotherapeutic agents (31). The difference in the IC50 of agents between the high and low NAD+ biosynthetic subtypes was evaluated with the Wilcoxon test, and *p*-value <0.05 was considered significant.

### Statistical and computational analysis

All statistical analyses were done in R (version 4.1). The significance of differences in continuous variables between the two subtypes was calculated using the Wilcoxon test. The Kruskal-Wallis test and Dunn’s *post hoc* multiple comparison test were used for more than two subtypes. The optimal cutoff values for each cohort were evaluated using the “surv_cutpoint” function in the “survminer” package. For prognostic analysis, the survival curves were constructed using the Kaplan-Meier method and log-rank test was used to judge differences between the subtypes. Spearman correlation analysis was carried out to determine the correlation coefficient. Statistical significance was considered as *p*-value <0.05.

## Results

### Identification of NAD+ biosynthetic subtypes and their correlation with biological functions in BRCA

**FIGURE 1:**
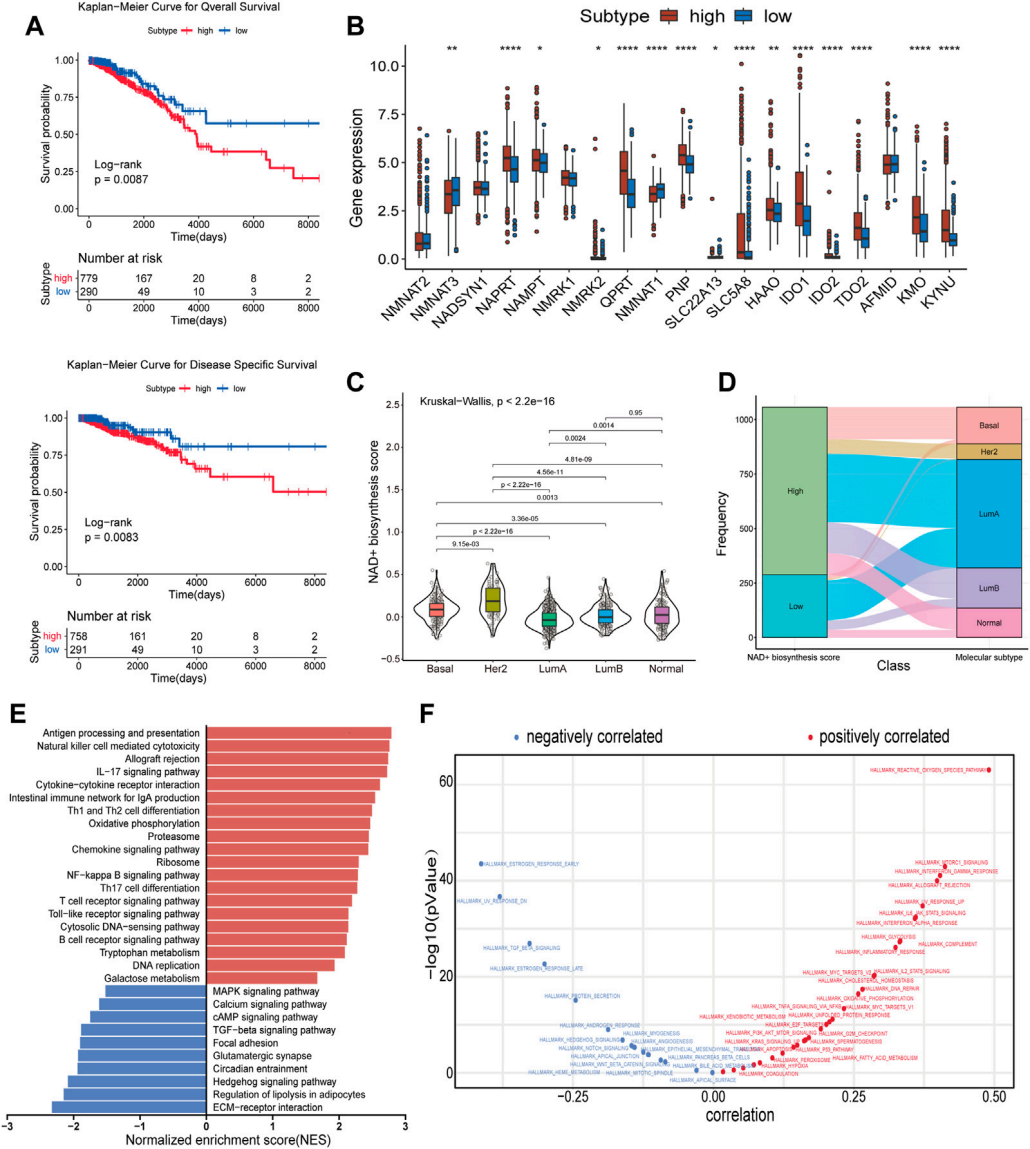
Subtypes of NAD+ biosynthesis and biological characteristics in BRCA. **(A)** Survival analysis of the high and low NAD+ biosynthetic subtypes in TCGA cohort. **(B)** Differential expression of NAD+ biosynthesis-related genes between the high and low NAD+ biosynthetic subtypes in TCGA cohort. **(C)** Violin plots showed the correlation between the NAD+ biosynthesis score and molecular subtypes in TCGA cohort. **(D)** Alluvial plot for the NAD+ biosynthetic subtypes versus different molecular subtypes in TCGA cohort. **(E)** Differential pathway activities scored by GSEA between the high and low biosynthetic subtypes in TCGA cohort. The red bars indicated the upregulated pathways, while the blue bars indicated the downregulated pathways. **(F)** Correlation between the NAD+ biosynthesis score and ssGSEA enrichment scores of cancer hallmark pathways in TCGA cohort. *p < 0.05; **p < 0.01; ***p < 0.001, and ****p < 0.0001.

NAD+ is essential for the proper function and metabolism of all living cells, including cancer cells. We used ssGSEA to calculate the NAD+ biosynthesis score of patients from TCGA database and found that NAD+ biosynthesis score of breast cancer tissue was significantly higher than that of normal breast tissue, which is consistent with previous findings (Supplementary Figure S1). To better understand the effect of NAD+ biosynthesis on breast cancer prognosis, the BC patients were divided into the high and low NAD+ biosynthetic subtypes based on the optimal cut-off value (−0.0743) in TCGA cohort, and subjected to survival comparison. Figure 1A showed that patients in the low NAD+ biosynthetic subtype had significantly better overall survival and disease specific survival than patients in the high NAD+ biosynthetic subtype. The prognostic significance of the NAD+ biosynthesis score was validated in GEO cohort (Supplementary Figure S2A). Compared to low NAD+ biosynthetic subtype, twelve NAD+ biosynthesis-related genes were significantly upregulated in the high NAD+ biosynthetic subtype, which indicated that NAD+ biosynthesis score may reflect the activity of NAD+ biosynthesis to some extent (Figure 1B). Next, we analyzed the clinical features differences between the two subtypes, including pathological stage, molecular subtypes and TNM stage (Supplementary Figure S3). We found that only molecular subtypes and NAD+ biosynthesis score exhibited statistical significance (Figures 1C, D). In detail, the HER2-enriched subtype had significantly higher NAD+ biosynthesis score than other four molecular subtypes, whereas the LumA subtype had the lowest NAD+ biosynthesis score.

GSEA was used to investigate the biological differences between the two subtypes. As shown in Figure 1E, the results showed that many immune-related pathways were significantly upregulated in the high NAD+ biosynthetic subtype, including antigen processing and presentation, IL-17 signaling pathway and chemokine signaling pathway. The low NAD+ biosynthetic subtype was enriched in pathways related to ECM-receptor interaction and regulation of lipolysis in adipocytes. Besides, to explore the potential pathways which NAD+ biosynthesis was involved in, the correlation between the NAD+ biosynthesis score and cancer hallmarks was analyzed. We found a significant positive association between the NAD+ biosynthesis score and some hallmark pathways, including reactive oxygen species, glycolysis, and DNA repair, which may explain the inconsistent clinical outcome between the two subtypes. We also found that the NAD+ biosynthesis score was positively correlated with immune-related pathways such as allograft rejection, IFN-γsignaling, inflammatory response. While pathways such as estrogen response, TGF-beta and hedgehog signaling were negatively correlated with the NAD+ biosynthesis score (Figure 1F). Similar biological differences between the two subtypes were observed in GEO cohort (Supplementary Figures S2C, D). Collectively, these findings suggested that NAD+ biosynthesis metabolism may play an important role not only in the development of breast cancer, but also in tumor immune environment.

### Immune landscape of NAD+ biosynthetic subtypes

**FIGURE 2:**
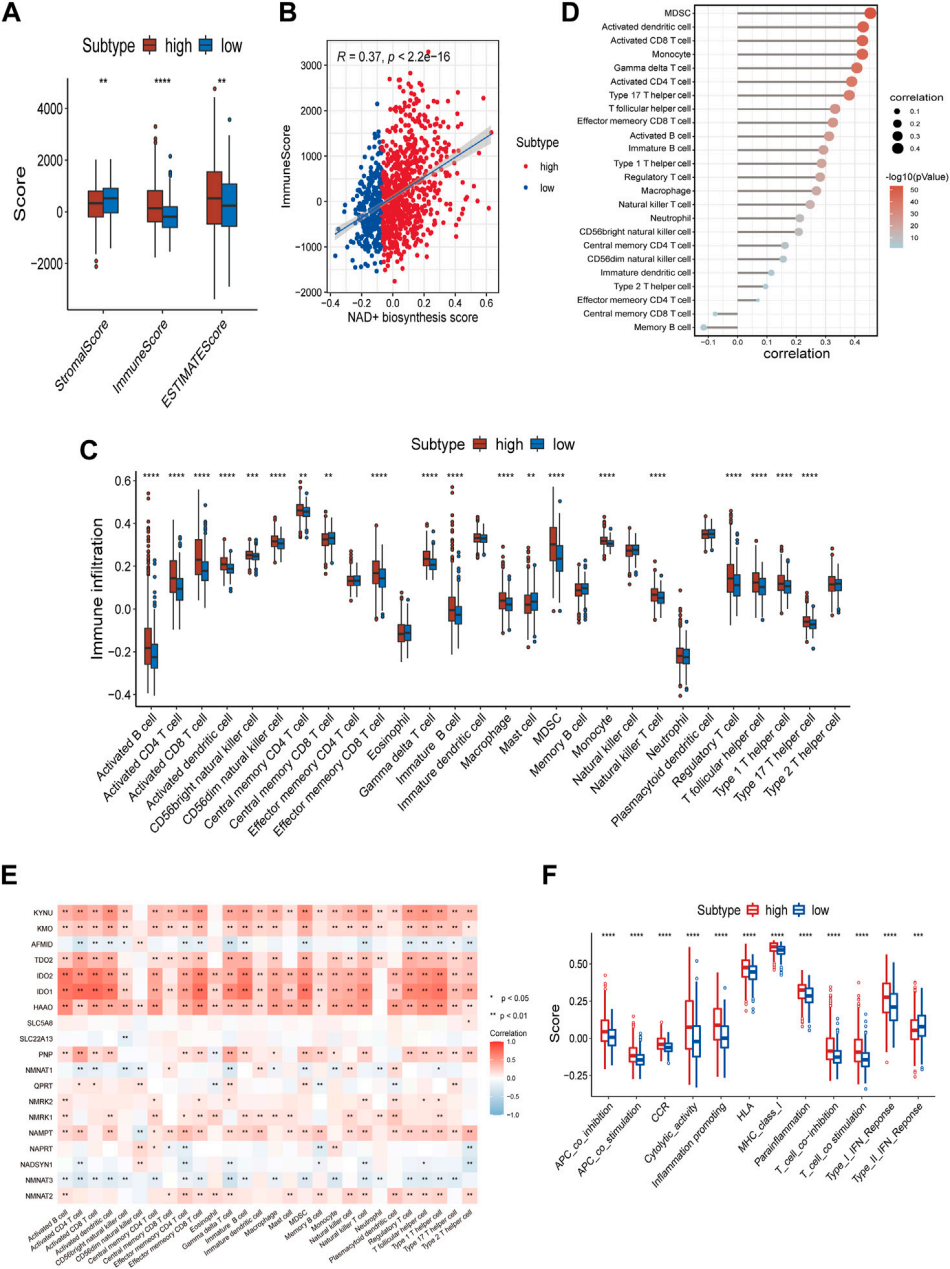
Correlation of the NAD+ biosynthesis score with the immune landscape of patients with breast cancer. **(A)** Comparison of the stromal score, immune score, and ESTIMATE score between the high and low NAD+ biosynthetic subtypes. **(B)** The correlation between the NAD+ biosynthesis score and immune score. **(C)** Relative infiltration of 28 types of immune cells in the high and low NAD+ biosynthetic subtypes. **(D)** The correlation between the NAD+ biosynthesis score and the ssGSEA enrichment scores of immune cells. **(E)** The heatmap showed the associations between the 19 NAD+ biosynthesis-related genes and immune cells. Red indicated positive correlations, and blue indicated negative correlations. Asterisks denoted *p*-value. Blank cells represented no statistical significance of the correlation. **(F)** Relative enrichment score of 12 immune-related signatures in the high and low NAD+ biosynthetic subtypes. *p < 0.05; **p < 0.01; ***p < 0.001, and ****p < 0.0001.

The state of TIME determines the fate of cancer cells. As indicated by the above results, NAD+ biosynthesis had a certain connection with immune response. As such, we assessed the differences in the immune microenvironment of patients in the two subtypes. The immune score, stromal score, and ESTIMATE score for the two subtypes were calculated using the ESTIMATE algorithm. The findings suggested that high NAD+ biosynthetic subtype had a higher ESTIMATE score, immune score, but a lower stromal score compared to the low NAD+ biosynthetic subtype (Figure 2A). We also found a significant positive correlation between the NAD+ biosynthesis score and immune score in breast cancer (Figure 2B), indicating that increased NAD+ biosynthesis was associated with immune activation and high immune infiltration in breast cancer. By comparing the ssGSEA score of 28 types of immune cells using the Wilcoxon test, we found that most immune cells, such as activated B cells, activated CD8^+^ T cells, activated dendritic cells, myeloid-derived suppressor cells (MDSCs) and regulatory T cells, were significantly enriched in the high NAD+ biosynthetic subtype, while few immune cells, such as central memory CD8 T cells and mast cells, were significantly enriched in the low NAD+ biosynthetic subtype (Figure 2C). The NAD+ biosynthesis score was observed closely associated with various categories of infiltrating immune cell, especially MDSCs (Figure 2D). The findings were similar in GEO cohort (Supplementary Figure S4). MDSCs are important components of TIME and can suppress the anti-tumor functions of T cells and natural killer cells (32). We believed that this immunosuppressive cell type may play an important role in the adverse clinical outcome of patients with high NAD+ biosynthesis score. The correlation analysis showed that most NAD+ biosynthesis-related genes, including NMRK1, PNP, IDO1 and NAMPT, were positively correlated with most immune cells, while negative correlations were demonstrated in molecules such as NMNAT2 and AFMID (Figure 2E). Moreover, the scores of most immune-related pathways were significantly higher in the high NAD+ biosynthetic subtype, including major histocompatibility complex (MHC) class I, and interferon response (Figure 2F). These results suggested that patients with high NAD+ biosynthetic activity tend to present a high immune infiltration and suppressive immune microenvironment.

### Potential TF-IRG regulatory network

**FIGURE 3:**
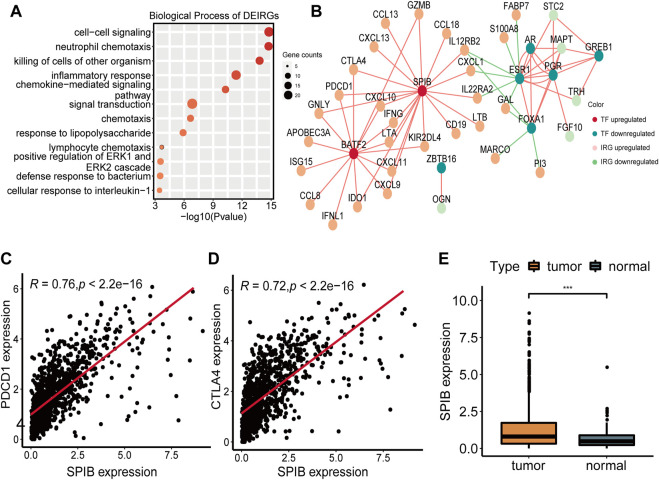
Construction of the TF-IRG network in BRCA. **(A)** GO enrichment analysis of 59 differentially expressed immune-related genes (DEIRGs). **(B)** A TF-IRG network in TCGA cohort. The pink line is the positive correlation between differentially expressed transcription factors (DETFs) and differentially expressed immune-related genes (DEIRGs). The green line is the negative correlation between DETFs and DEIRGs. **(C)** The correlation between the expression of SPIB and PDCD1 in TCGA cohort. **(D)** The correlation between the expression of SPIB and CTLA4 in TCGA cohort. **(E)** Differential expression of SPIB between breast normal and tumor tissues.

To further explore the potential regulatory mechanism of NAD+ biosynthesis affecting TIME, we identified 59 differentially expressed immune-related genes (DEIRGs), including 45 upregulated DEIRGs and 14 downregulated DEIRGs, which may play a major role in regulating TIME. DEIRGs are strongly associated with chemokine-mediated signaling (Figure 3A). Enhanced expression levels of T-cells recruiting chemokines such as CXCL9, CXCL10, and CXCL11 were found in the high NAD+ biosynthetic subtype, consistent with their abundant T cells infiltration. Additionally, the overexpression of CXCL13 (which can recruit MDSC) and other tumor suppressive chemokines (e.g., CCL18, CCL8 and CCL20) was also observed in the high NAD+ biosynthetic subtype (Supplementary Table S2). Transcriptional factors (TFs) can control the expression of critical genes and thus play an important role in the regulation of tumor inflammation and immunity (33, 34). To explore the potential upstream regulatory mechanism of DEIRGs, 11 differentially expressed TFs (DETFs) were also found from DEGs (Supplementary Table S3). A TF-IRG network was constructed based on the correlation of gene expression between DETFs and DEIRGs. Only gene pairs with correlation coefficients >0.5 (or less than −0.5) and adjusted *p*-value <0.01 were incorporated into the network (Supplementary Table S4). The TF-IRG network was visualized in Cytoscape (Figure 3B). We found that two TF genes, BATF2 and SPIB were significantly elevated in the high NAD+ biosynthetic subtype. Notably, SPIB was significantly associated with PDCD1 and CTLA4 (Figures 3C, D). The relationships between SPIB, PDCD1 and CTLA4 were also verified in GEO cohort (Supplementary Figure S5B). SPIB has been reported to play a negative role in immune cell regulation (35). Therefore, the SPIB-centered regulatory network may play an important role in immune infiltration and immune escape in the high NAD+ biosynthetic subtypes. We further confirmed that the expression level of SPIB was significantly higher in tumor tissues than in adjacent normal tissues (Figure 3E).

### Mutation and immunotherapy sensitivity prediction of NAD+ biosynthetic subtypes

**FIGURE 4:**
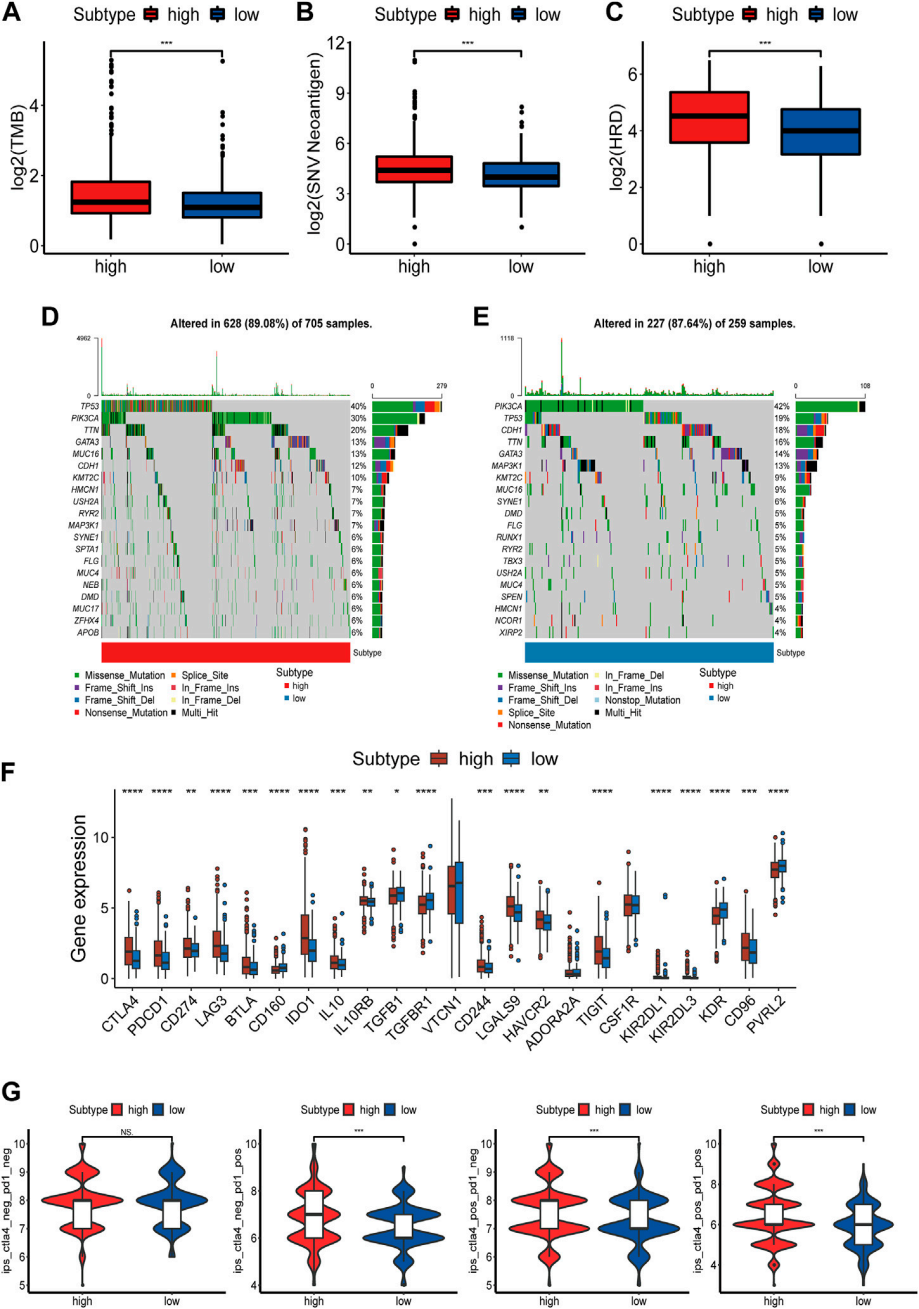
Mutation and Immunotherapy Sensitivity Prediction of the NAD+ biosynthesis signature. **(A–C)** TMB, Neoantigen and HRD between the high and low NAD+ biosynthetic subtypes. **(D,E)** Waterfall plot of tumor somatic mutation in the high and low NAD+ biosynthetic subtypes. **(F)** Comparison of immune checkpoints expression levels between the high and low NAD+ biosynthetic subtypes. **(G)** Comparison of the immunophenoscore (IPS) between the high and low NAD+ biosynthetic subtypes. *p < 0.05; **p < 0.01; ***p < 0.001, and ****p < 0.0001.

Immunotherapy has become an emerging clinical strategy for treating cancer (36). Considering that enhanced tumor immunogenicity predicts long-term clinical benefits for patients from immune checkpoint inhibitors (ICIs), we wanted to explore the relationship between NAD+ biosynthesis scores and tumor immunogenicity. Therefore, we took the perspective of the tumor mutational burden (TMB), neoantigen load and homologous recombination deficiency (HRD). Results showed that patients in the high NAD+ biosynthetic subtype had higher TMB, neoantigen and HRD (Figures 4A–C). The subsequent correlation analysis showed that TMB, neoantigen load and HRD were positively correlated with the NAD+ biosynthesis score (Supplementary Figure S6). These results suggested that patients in the high NAD+ biosynthetic subtype had relatively high immunogenicity. Somatic mutation analysis revealed higher TP53 mutation rates in the high NAD+ biosynthetic subtype and higher PIK3CA mutation rates in the low NAD+ biosynthetic subtype. (Figures 4D, E). Additionally, the expression of immune checkpoint molecules such as PD-L1, LAG3, PD-1, and CTLA-4 was higher in the high NAD+ biosynthetic subtype (Figure 4F) We also assessed the association between IPS and NAD+ biosynthesis score. The results illustrated that the IPS for PD1/PD-L1/PD-L2 blocker, CTLA-4 blocker and PD1/PD-L1/PD-L2 plus CTLA-4 blocker in high NAD+ biosynthetic subtype were significantly higher than those in low NAD+ biosynthetic subtype (Figure 4G). These findings suggested that patients with high NAD+ biosynthetic score may be appropriate candidates for immunotherapy.

### Drug sensitivity prediction

**FIGURE 5:**
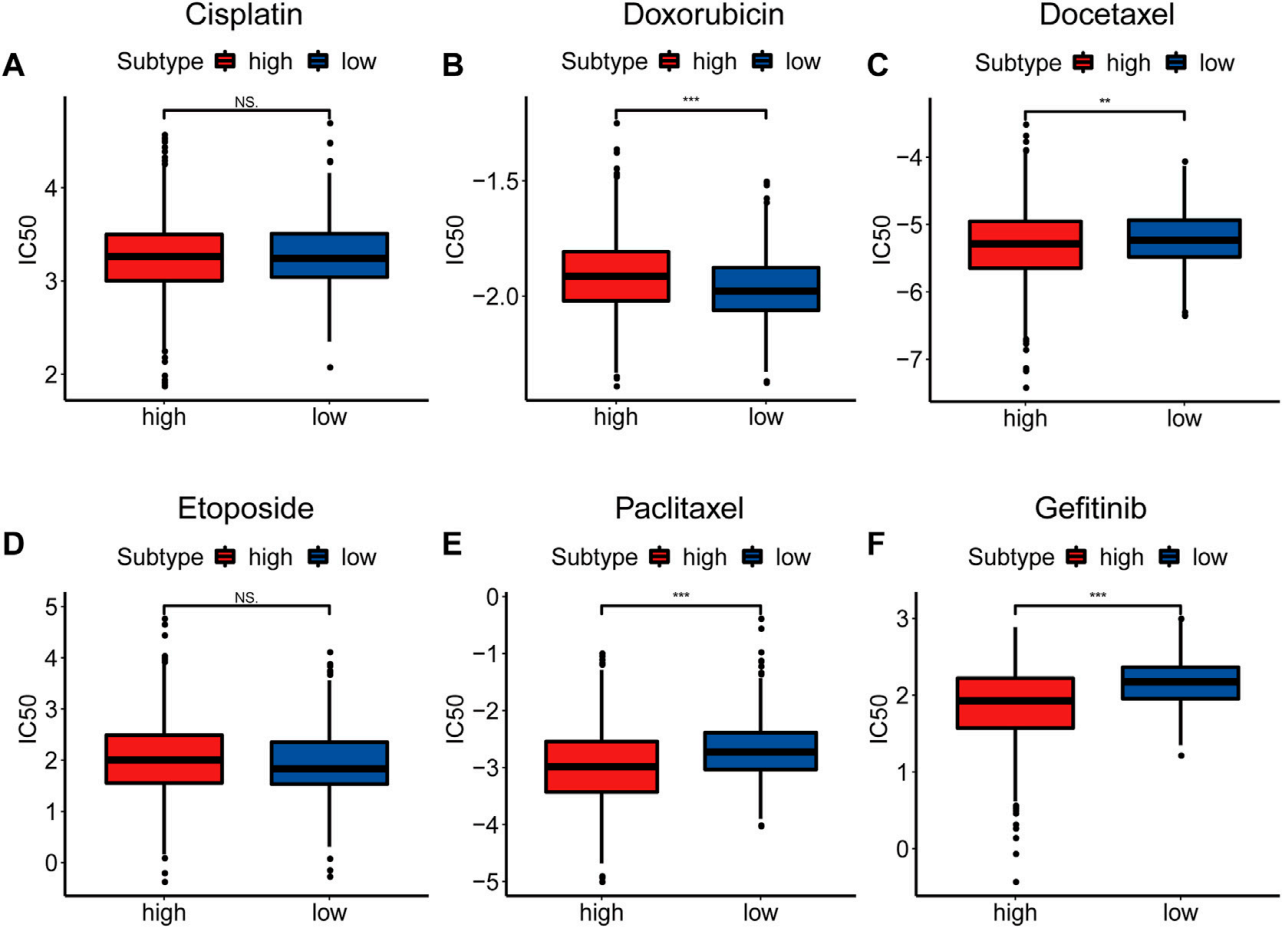
Drug Sensitivity Prediction. **(A–F)** Box plots of the estimated IC50 for Cisplatin (A), Doxorubicin (B), Docetaxel (C), Etoposide(D), Paclitaxel (E) and Gefitinib (F). *p < 0.05; **p < 0.01; ***p < 0.001.

Considering that drug chemosensitivity can influence the clinical outcomes of breast cancer treatment, we predicted the IC50 of usual chemotherapeutic drugs and compared them between the two subtypes. Results showed that the estimated IC50 for doxorubicin was lower in the low NAD+ biosynthetic subtype, while the estimated IC50s for docetaxel, paclitaxel and gefitinib were lower in the high NAD+ biosynthetic subtype. However, there was no significant difference in the estimated IC50s for cisplatin and etoposide (Figures 5A–F). These results suggested that the NAD+ biosynthesis score may differentiate more patients for appropriate therapy.

## Discussion

The role of NAD+ in cellular metabolism and signal transduction is crucial as it cannot be obtained directly from food, making NAD+ biosynthesis essential (12). Previous works have shown that NAD+ is critical for the development and progression of breast cancer. For example, QPRT, one of the rate-limiting enzymes of NAD+ biosynthesis, was shown to potentially enhance breast cancer invasiveness through purinergic signaling (37). However, little work has explored the relationship between NAD+ biosynthesis metabolism and TIME in breast cancer. Thus, in this study, we quantified NAD+ biosynthesis metabolism based on ssGSEA and demonstrated that the NAD+ biosynthesis score was significantly associated with the overall survival. Further bioinformatics analyses revealed that the NAD+ biosynthesis score was significantly related to different tumor-infiltrating immune cells and other immune-related signatures. Interestingly, we also found that the NAD+ biosynthesis score was positively associated with several immunotherapy biomarkers. This indicates that patients in the high NAD+ biosynthesis subtype may be more suitable for immunotherapy. As far as we know, this study is the first study to systematically explore the relationship between NAD+ biosynthesis metabolism and TIME in breast cancer.

In this study, we divided patients with breast cancer into the high and low biosynthetic subtypes based on the NAD+ biosynthesis score. We found that high NAD+ biosynthetic subtype was associated with poor prognosis. This correlation was validated in GEO cohort. GSEA analysis revealed that the NAD+ biosynthesis score was positively correlated with immune-related biological processes and pathways, suggesting that the difference in survival between the two subtypes may be due to immune heterogeneity. Then, analysis of the immune microenvironment showed that high NAD+ biosynthetic subtype was characterized by immune activation and accompanied with immune suppression. The ssGSEA scores of most immune cells in high NAD+ biosynthetic subtypes were higher than those in low NAD+ biosynthetic subtypes, which indicated that the immune microenvironment in BC with high biosynthesis score tended to be “hot.” Our results also showed that HER2-enriched and Basal subtypes had higher NAD+ biosynthesis score, which were consistent with higher TILs infiltration in the two molecular subtypes (38). However, high NAD+ biosynthetic subtype did not display a corresponding survival advantage and we found MDSC might play a key role. MDSC could promote immune evasion, tumor angiogenesis and metastasis (39). Besides, there is a positive association between IFN-γ signaling and NAD+ biosynthesis score. IFN-γ has a dual role. On the one hand, IFN-γ can enhance anticancer activity by increasing MHC class I and cytotoxic proteins associated with the CTL response. On the other hand, it also promotes the expression of PD-L1, CTLA-4, IDO1, CXCL12 and other molecules to promote tumor immune escape(40, 41). In fact, it has been reported that NAD+ could enhance IFN-γ-induced PD-L1 expression and promote tumor immune evasion (42). Therefore, we believe that IFN-γ signaling and MDSC could contribute to the immunosuppressive microenvironment in NAD+ biosynthesis-activated breast cancer.

Besides, the TF-IRG regulatory network implied that two transcription factors, BATF2 and SPIB, may play an active role in transcriptional events associated with the infiltration of immune cells in the high NAD+ biosynthetic subtype. BATF2, a member of the basic leucine zipper transcription factor family, can be involved in the gene regulation of IFN-γ-activated classical macrophages and induce pro-inflammatory responses (43, 44). SPIB, belongs to the ETS family, plays an important role in the differentiation of B cells and plasmacytoid dendritic cells (45, 46). SPIB has been shown to promote tumor aerobic glycolysis (47) and participate in the recruitment of tumor-associated macrophages (TAMs) (35), which promotes cancer progression. In conclusion, the construction of the TF-IRG network provided some insights into the interaction between NAD+ biosynthesis and TIME in breast cancer.

Immunotherapy, represented by immune checkpoint inhibitors (ICIs), is changing the treatment of cancer. The higher immunogenicity in the high NAD+ biosynthetic subtype may lead to a better prognosis after receiving immunotherapy (48). TMB is a more reliable biomarker for predicting immunotherapy efficacy. Patients with high TMB will achieve higher objective response rates to immunotherapy (49, 50). The positive association between HRD and TMB has been reported in BC (51). The underlying mechanism for this correlation is that HRD may drive tumorigenesis, increasing the number of tumor mutations and the neoantigen rate in BC (52, 53). Neoantigens can promote CD8^+^ T cell infiltration and cytolytic activity, which are closely associated with response to immunogenicity. Mutation analysis showed high NAD+ biosynthesis was associated with high TP53 mutation rate. One study reported that patients with mutant TP53 showed stronger tumor antigenicity and tumor antigen presentation and were more likely to benefit from immunotherapy (54), which was consistent with our findings. Moreover, we also found that patients with different NAD+ biosynthesis scores might exhibit distinct sensitivity to doxorubicin, docetaxel, paclitaxel, and gefitinib. These results suggested that NAD+ biosynthesis score can assess the efficacy of immunotherapy and chemotherapy, facilitating personalized treatment for breast cancer.

This study has some limitations. Firstly, in our study, the clinical cohorts were derived from public databases and need further validated by prospective studies. Secondly, the association between NAD+ biosynthesis and TIME requires additional experimental validation.

In conclusion, our study suggests that NAD+ biosynthetic activity was correlated with the prognosis of breast cancer and the immune microenvironment. NAD+ biosynthetic activity may also serve as a potential prognostic biomarker for ICI immunotherapy, which may help to screen patients eligible for immunotherapy and guide future individualized precision therapy.

## Data Availability

The public datasets to support the results of this research can be gained from TCGA (https://portal.gdc.cancer.gov/) and GEO (https://www.ncbi.nlm.nih.gov/geo/).
