# Supplementary material for: NAD+ biosynthesis metabolism predicts prognosis and indicates immune microenvironment for breast cancer
Source: Pathol Oncol Res. 2023 Mar 17;29:1610956. doi: 10.3389/pore.2023.1610956 (PMC10063816; doi:10.3389/pore.2023.1610956)
Supplement: Supplementary file 1 [file DataSheet1.ZIP › 补充文件/Supplementary Figure.docx]

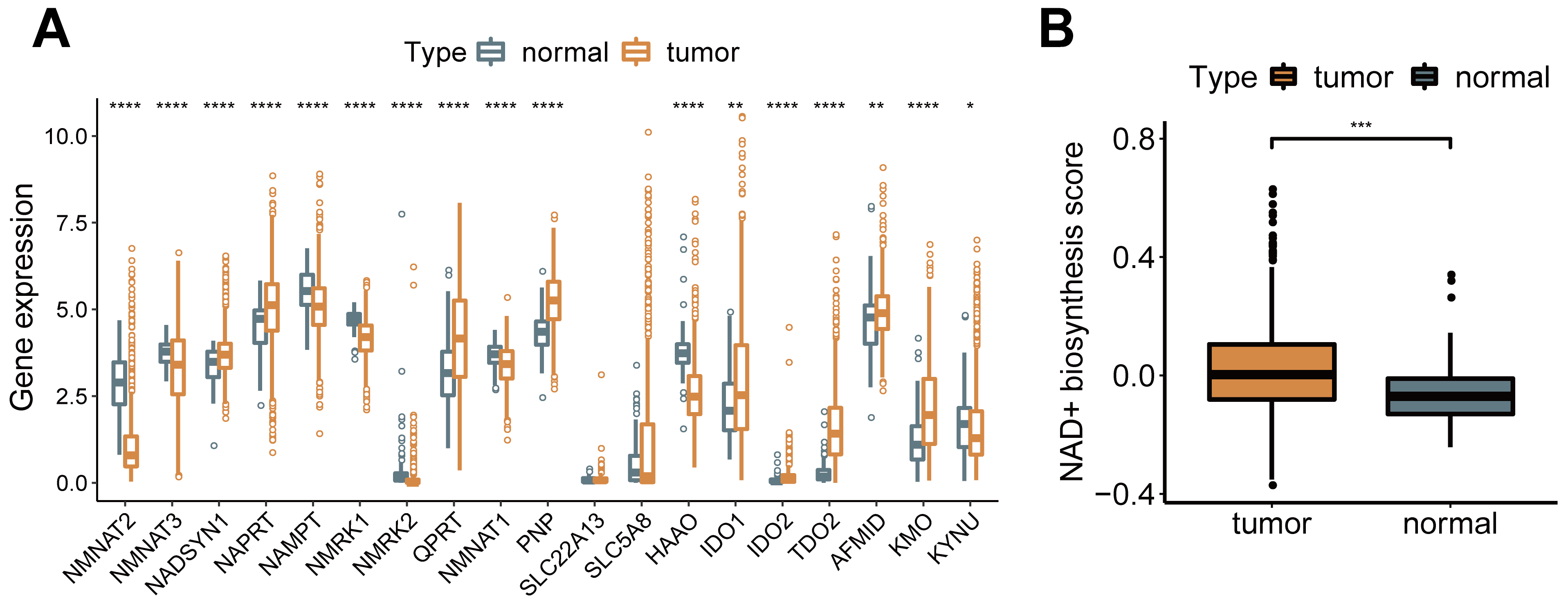
Supplementary Figure 1. Comparison of NAD+ biosynthesis signature between normal and tumor tissues. (A) Boxplots showed differential expression of NAD+ biosynthesis-related genes between normal and tumor tissues. (B) Comparison of the NAD+ biosynthesis score between normal and tumor tissues.*p < 0.05; **p < 0.01; ***p < 0.001, and ****p < 0.0001.


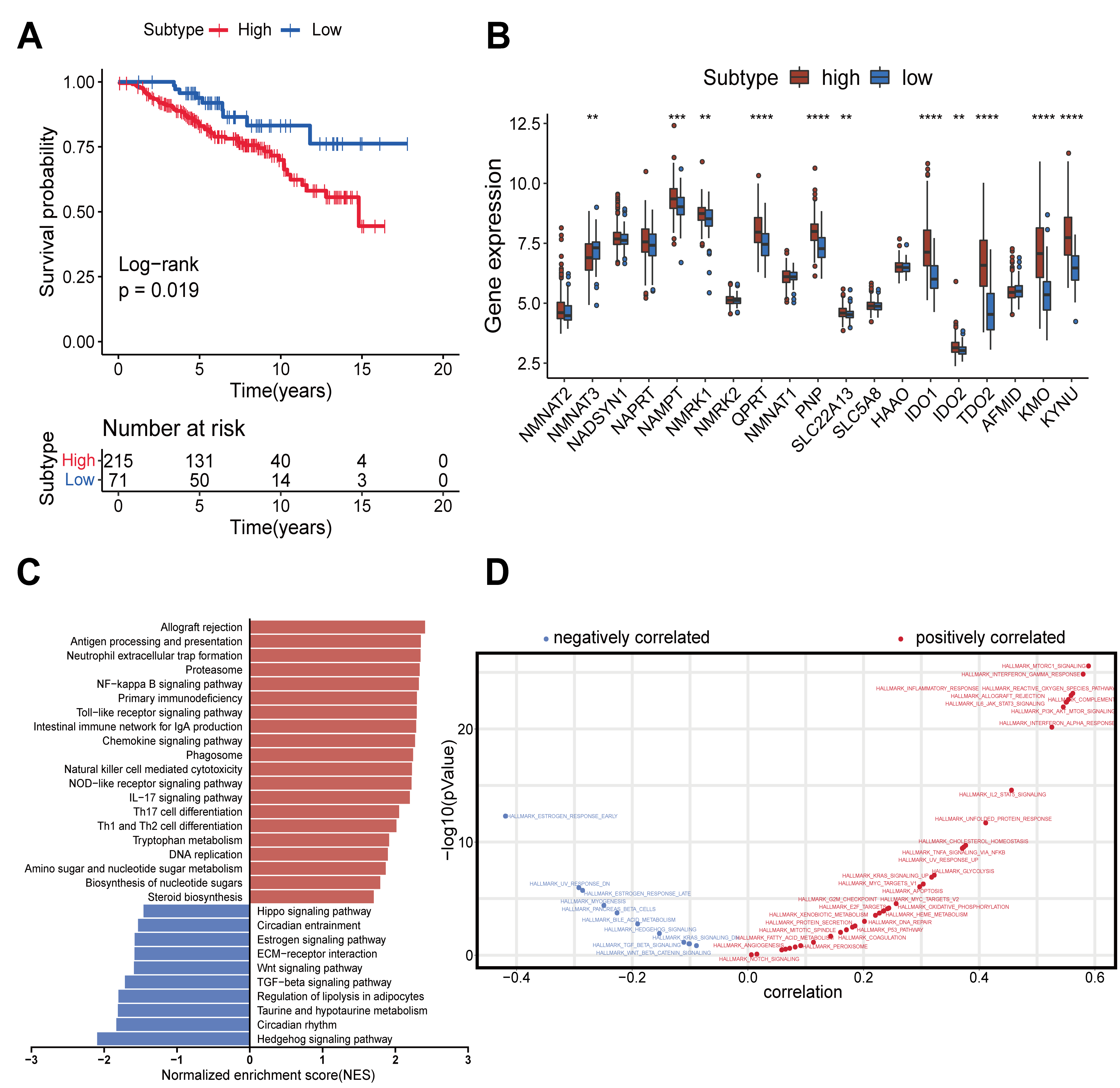


Supplementary Figure 2. Subtypes of NAD+ biosynthesis score and biological characteristics in GEO cohort. (A) Kaplan–Meier survival analysis of the high and low NAD+ biosynthetic subtypes in GEO cohort. (B) Boxplots showed differential expression of NAD+ biosynthesis-related genes between the high and low NAD+ biosynthetic subtypes. (C) Differential pathway activities scored by GSEA between the high and low biosynthetic subtypes in GEO cohort. The red bars indicated the upregulated pathways, while the blue bars indicated the downregulated pathways. (D) Correlation between the NAD+ biosynthesis score and ssGSEA enrichment scores of cancer hallmark pathways in GEO cohort. *p < 0.05; **p < 0.01; ***p < 0.001, and ****p < 0.0001.


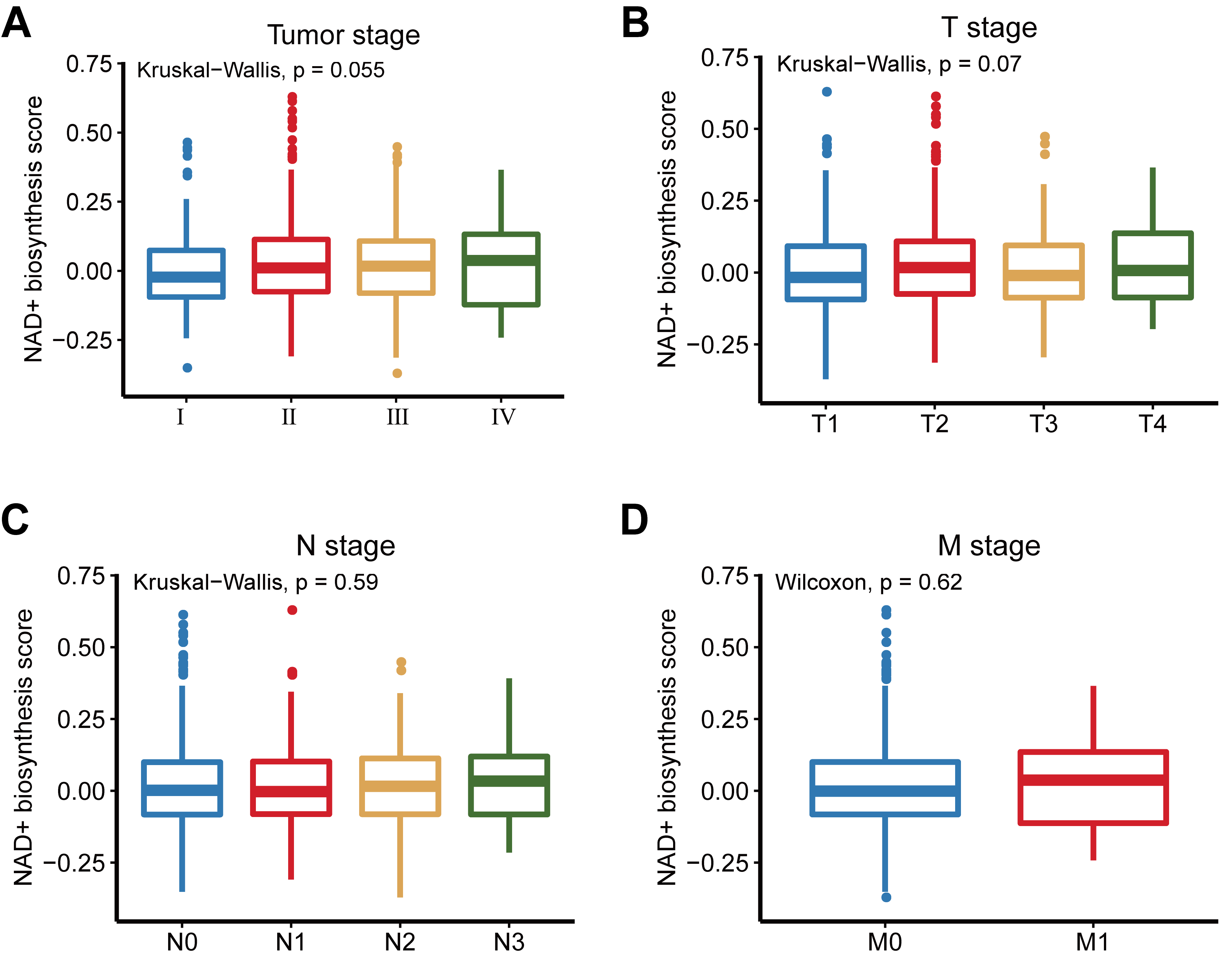


Supplementary Figure 3. The association between the NAD+ biosynthesis score and clinical features. The NAD+ biosynthesis score distribution in patients with different clinical features including tumor stage (A), and T stage (B), N stage (C) and M stage (D).


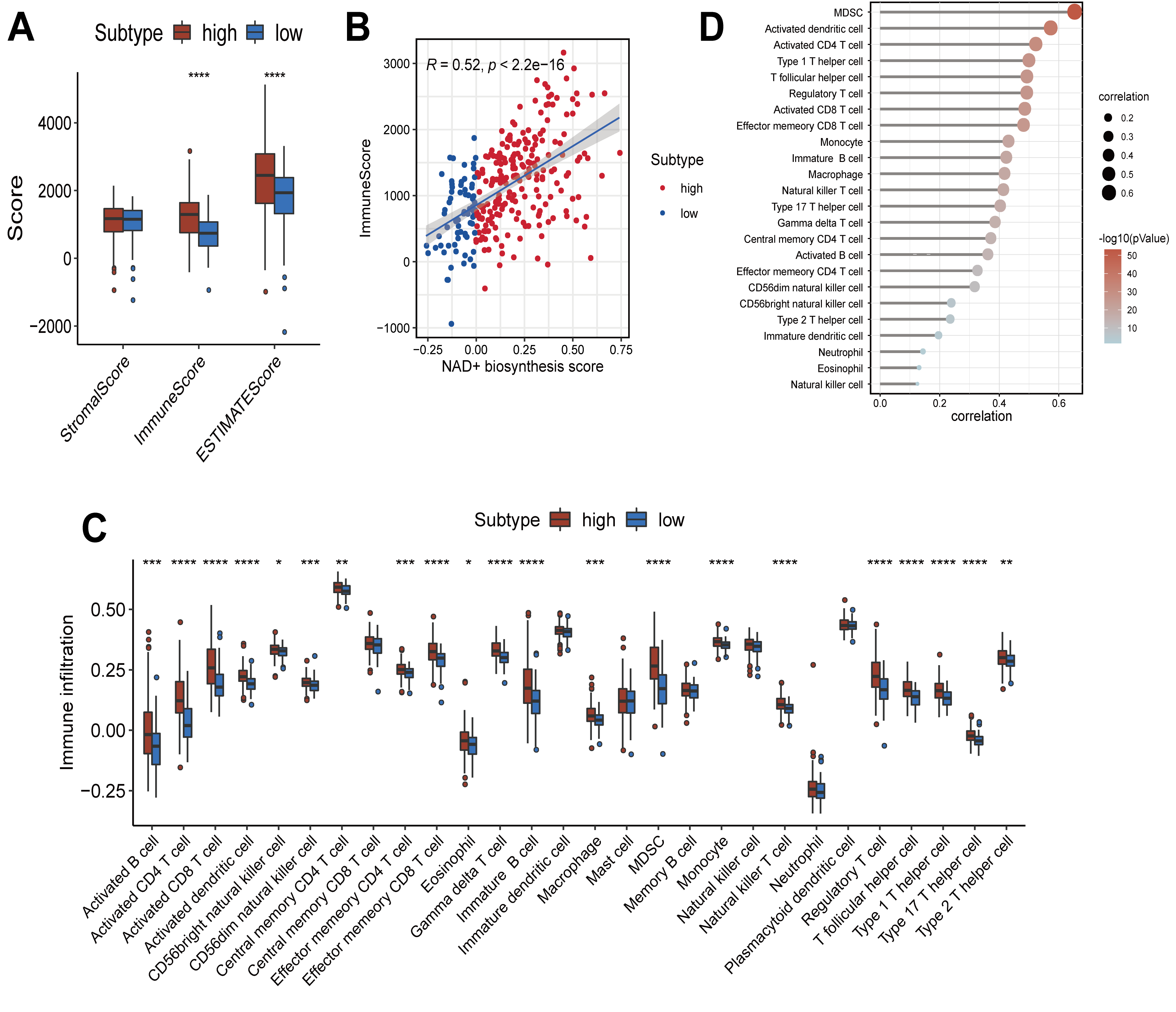
Supplementary Figure 4. Correlation of the NAD+ biosynthesis score with the immune landscape of patients with breast cancer in GEO cohort. (A) Differences in the stromal score, immune score, and ESTIMATE score between the high and low NAD+ biosynthetic subtypes. (B) The correlation between the NAD+ biosynthesis score and immune score. (C) Relative infiltration of 28 types of immune cells in the high and low NAD+ biosynthetic subtypes. (D) The correlation between the NAD+ biosynthesis score and the ssGSEA enrichment scores of immune cells in GEO cohort. *p < 0.05; **p < 0.01; ***p < 0.001, and ****p < 0.0001.


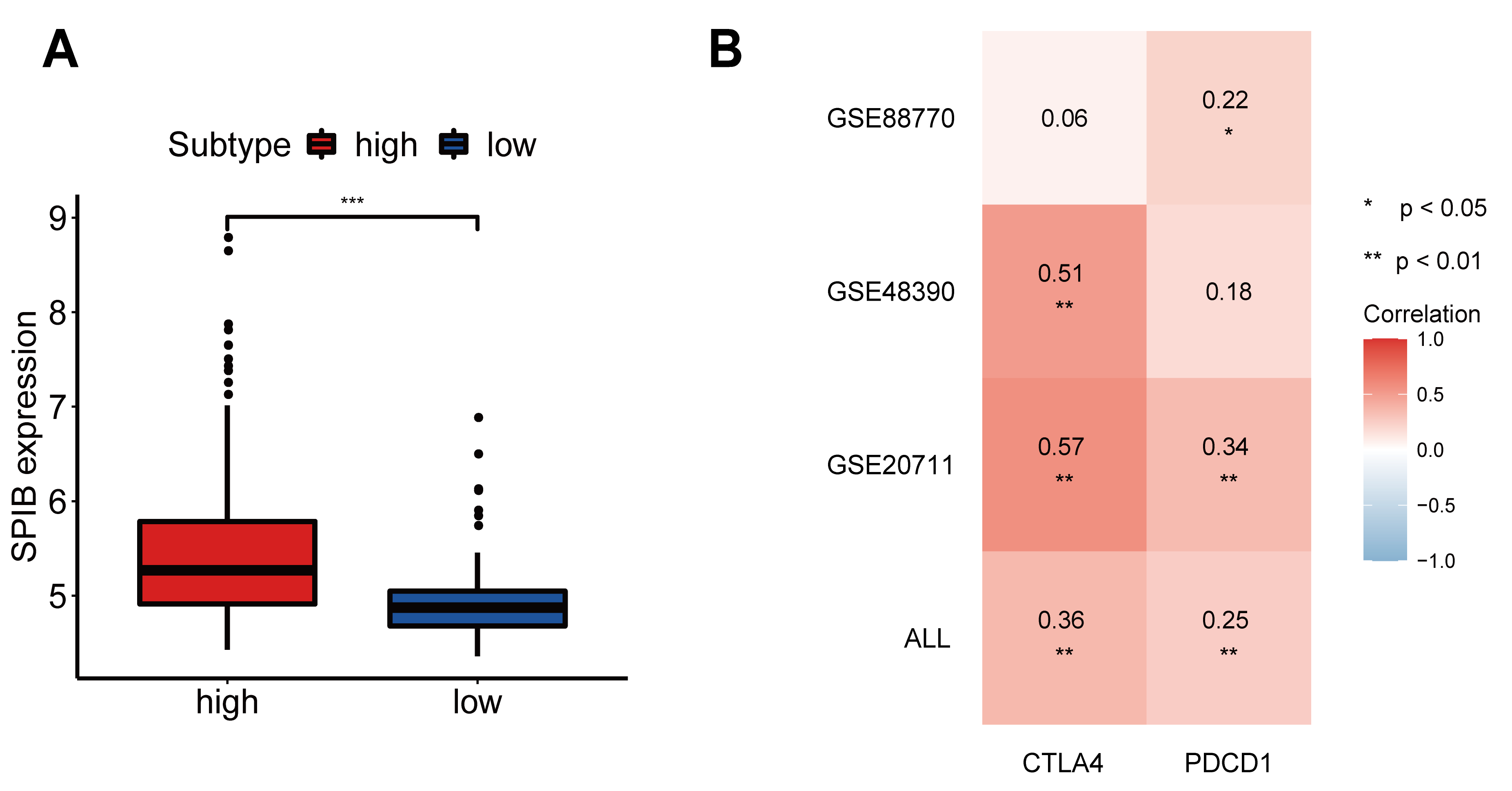


Supplementary Figure 5. Expression of SPIB in GEO cohort. (A) Differential expression of SPIB between the high and low NAD+ biosynthetic subtypes. (B) The correlation between the expression of SPIB and PDCD1 and CTLA4 in GEO cohort, including GSE88770, GSE48390 and GSE20711.


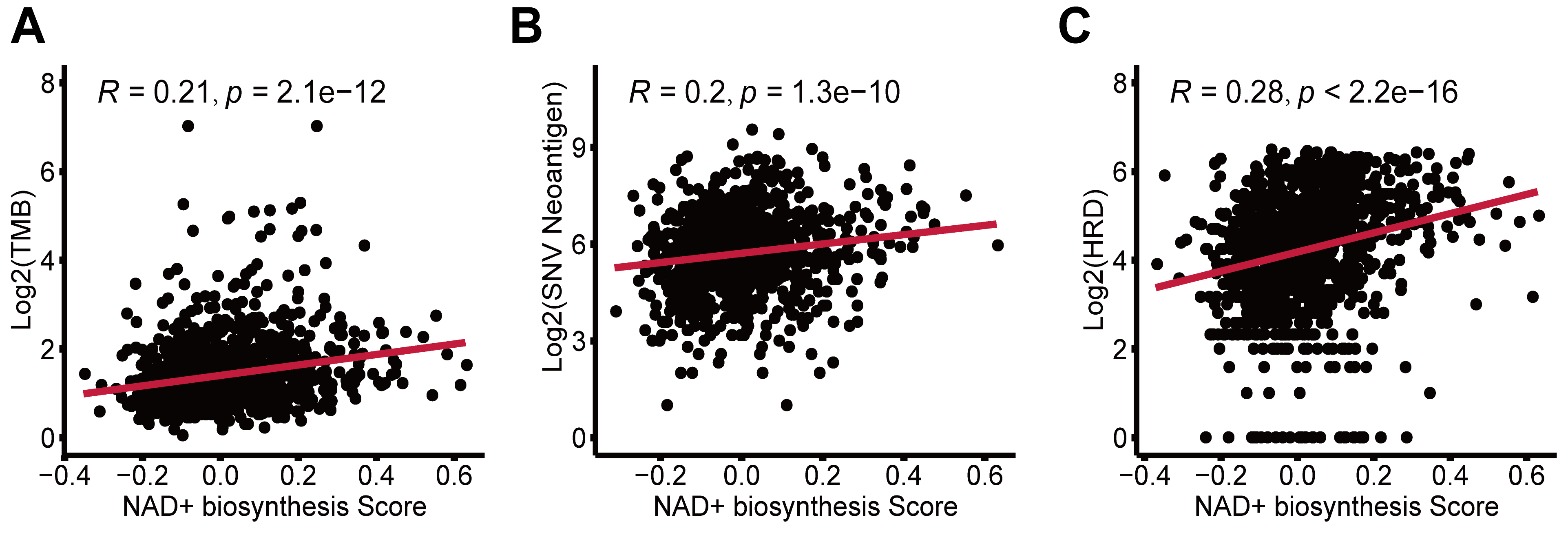


Supplementary Figure 6. Association between the NAD+ biosynthesis score and immunogenicity in TCGA cohort. The positive correlation between the NAD+ biosynthesis score and TMB (A) , Neoantigen (B) and HRD (C).
